# Workload-indexed blood pressure response to exercise: considerations for future studies estimating maximal oxygen uptake

**DOI:** 10.1093/eurjpc/zwae071

**Published:** 2024-02-29

**Authors:** Alise D Rycroft, Sydney E Hilton, Pardeep K Khangura, Julian C Bommarito, Massimo Nardone, Philip J Millar

**Affiliations:** Human Cardiovascular Physiology Laboratory, Department of Human Health and Nutritional Sciences, University of Guelph, ANNU 348A, 50 Stone Road East, Guelph, Ontario N1G2W1, Canada; Human Cardiovascular Physiology Laboratory, Department of Human Health and Nutritional Sciences, University of Guelph, ANNU 348A, 50 Stone Road East, Guelph, Ontario N1G2W1, Canada; Human Cardiovascular Physiology Laboratory, Department of Human Health and Nutritional Sciences, University of Guelph, ANNU 348A, 50 Stone Road East, Guelph, Ontario N1G2W1, Canada; Human Cardiovascular Physiology Laboratory, Department of Human Health and Nutritional Sciences, University of Guelph, ANNU 348A, 50 Stone Road East, Guelph, Ontario N1G2W1, Canada; Human Cardiovascular Physiology Laboratory, Department of Human Health and Nutritional Sciences, University of Guelph, ANNU 348A, 50 Stone Road East, Guelph, Ontario N1G2W1, Canada; Human Cardiovascular Physiology Laboratory, Department of Human Health and Nutritional Sciences, University of Guelph, ANNU 348A, 50 Stone Road East, Guelph, Ontario N1G2W1, Canada

Hypertension is the leading chronic risk factor for worldwide mortality.^1^ In normotensive adults, one early warning sign for the future development of hypertension is an exaggerated blood pressure response (EBPR) during submaximal or peak exercise.^2^ One concern regarding the utility of peak exercise blood pressure has been the relationship with aerobic fitness, such that those with a higher absolute maximal oxygen consumption (VO_2_peak) are associated with a higher peak exercise systolic blood pressure (SBP).^3^ Hedman *et al.*^4^ recently reported that peak SBP normalized to workload was a better predictor of all-cause mortality than peak SBP during maximal treadmill exercise in a large cohort of male veterans. Workload was based on metabolic equivalents of task (MET) and estimated using an equation (VO_2_ max = (speed [m/min] × 0.2) + (speed [m/min] × grade [%] × 0.9) + 3.5) developed by the American College of Sports Medicine (ACSM).^5^ Following this finding, several small prospective studies have adopted similar methods of calculating the SBP/MET slope.^6,7^ However, to our knowledge, no study has examined whether the SBP/MET slope calculated based on the ACSM equation for maximal treadmill exercise is consistent in identifying those with an at-risk phenotype compared with direct measures of oxygen consumption using indirect calorimetry. We tested the hypothesis that the SBP/MET slope during treadmill exercise would differ when calculated using the ACSM or the Fitness Registry and Importance of Exercise Database {FRIEND; VO_2_ max = 79.9 − (0.39 × age) − (13.7 × sex [0 = male, 1 = female]) − (0.127 × weight [lb])} equations for predicted maximal oxygen uptake compared with direct measurements of oxygen consumption.

We studied 63 otherwise healthy males and females between the ages of 18 and 40 years, who were recruited prospectively as part of an ongoing study on mechanisms contributing to an exercise EBPR. All participants completed the Physical Activity Readiness Questionnaire (PAR-Q+) and self-reported to be free of any cardiometabolic disease or chronic medications, apart from oral contraceptives. This study complies with the Declaration of Helsinki and was approved by our institutional research ethics board (REB#206027). Informed written consent was obtained from all participants before commencing the study.

Participants completed one visit to the laboratory following a 24-h abstention from alcohol, recreational drugs, caffeine, and strenuous exercise and 3-h post-prandial. Each participant completed a maximal treadmill exercise using the standardized Bruce protocol. Before the exercise test, participants were given 5 min of quiet rest followed by six discrete measurements of brachial blood pressure and heart rate (BPM-200, BpTRU, Coquitlam, BC, Canada). Blood pressure was also collected when participants were standing on the treadmill at rest and every 90 s throughout exercise (Tango M2, SunTech Medical Inc., NC, USA). Breath-by-breath oxygen consumption was analysed (Cosmed Quark CPET, Rome, Italy) and filtered using a 30-s rolling average to calculate MET levels during exercise. Peak SBP was defined as the highest reading collected during the exercise protocol. In accordance with prior work,^4^ SBP/MET slope was defined as the difference between the peak and standing blood pressure divided by the MET value (coincident with the peak SBP) minus 1. Estimated MET levels were computed using both ACSM and FRIEND equations.^5,8^ Statistical analyses were performed using GraphPad Prism 9.4.0 (GraphPad Software, San Diego, CA, USA). Due to non-normal distributions (Shapiro–Wilk test), the Friedman test was used to compare collected VO_2_peak vs. estimated VO_2_peak equations and corresponding SBP/MET slopes. Fisher’s exact tests were performed to examine the proportion of participants identified as having an SBP/MET slope >9 mmHg/MET or below. This cut-off was selected as a SBP/MET slope of >9 mmHg/MET has been associated with a greater risk of all-cause mortality in males.^4^ The sensitivity and specificity for identifying a SBP/MET slope >9 mmHg/MET were calculated. Unpaired *t*-tests were used to compare between male and female participants. Statistical significance was considered *P* < 0.05.

Baseline participant characteristics are listed on *Table 1*. Participants were young and normotensive, with a wide range in cardiorespiratory fitness. The sample was 43% female, with almost ∼40% identifying as Black, Asian, or Indigenous. Collected VO_2_peak was lower than VO_2_peak estimated using the ACSM equation (*P* = 0.03) but not different from VO_2_peak estimated using the FRIEND equation (*P* > 0.99; *Figure 1A*). Estimated VO_2_peak also differed between ACSM and FRIEND equations (*P* < 0.001). As a result, the SBP/MET slopes calculated from collected VO_2_ data and the FRIEND equation were both higher than the SBP/MET slopes calculated using the ACSM equation (*P* < 0.001; *Figure 1B*). No statistical differences were found between the SBP/MET slopes calculated using collected VO_2_ data and the FRIEND equation (*P* = 0.98). These comparisons were similar when males and females were examined separately (data not shown). The proportion of participants identified as having an SBP/MET slope >9 mmHg/MET differed when using collected VO_2_ data vs. the ACSM equation (*P* < 0.02; *Figure 1C*), but not when collected VO_2_ data were compared with the FRIEND equation (*P* > 0.99; *Figure 1D*). ACSM and FRIEND equations both demonstrated high specificity, 100 and 90%, respectively, and low sensitivity, 18 and 45%, respectively.

**Figure 1:**
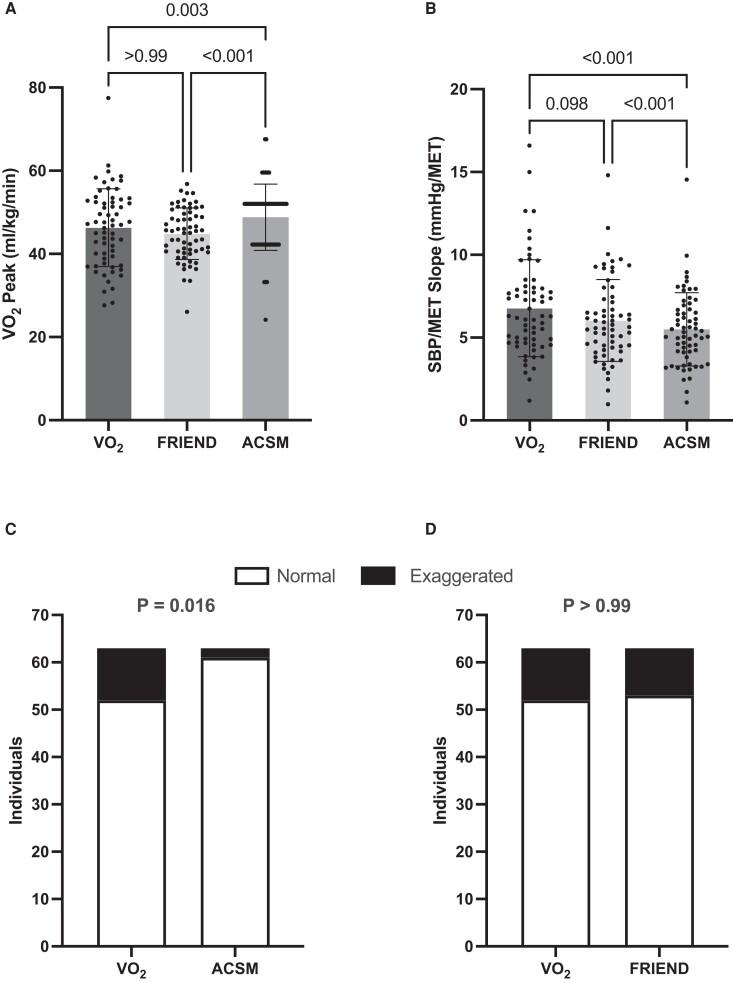
Comparison of relative VO_2_peak (*A*) and the systolic blood pressure/metabolic equivalent of task slope (*B*) using oxygen consumption measured using indirect calorimetry or estimated using American College of Sports Medicine and Fitness Registry and Importance of Exercise Database equations. Comparison of the number of individuals identified as having a systolic blood pressure/metabolic equivalent of task slope >9 mmHg per metabolic equivalent of task using indirect calorimetry vs. American College of Sports Medicine (*C*) or indirect calorimetry vs. Fitness Registry and Importance of Exercise Database (*D*).

**Table 1:** Baseline participant demographics and characteristics

	Total (*n* = 63)	Male (*n* = 36)	Female (*n* = 27)	*P*-value
Black (%)	12.7%	8.3%	18.5%	0.27
Caucasian (%)	60.3%	61.1%	59.3%	>0.99
Asian (%)	23.8%	27.8%	18.5%	0.55
Indigenous (%)	3.2%	2.8%	3.7%	>0.99
Age (years)	23 ± 6 (18–40)	24 ± 6 (18–39)	22 ± 6 (18–40)	0.19
Resting SBP (mmHg)	106 ± 9 (88–124)	110 ± 7 (94–124)	100 ± 8 (88–116)	<0.01
Resting DBP (mmHg)	68 ± 7 (51–84)	69 ± 8 (51–84)	67 ± 6 (57–75)	0.23
VO_2_peak (mL/kg/min)	46.3 ± 9 (27.6–77.5)	50.2 ± 9 (30.9–77.5)	41.2 ± 7 (27.6–53.1)	<0.01
BMI (kg/m^2^)	23.8 ± 4 (16.9–34.6)	24.7 ± 4 (17.9–34.6)	22.5 ± 4 (16.9–33.3)	0.02
Peak SBP (mmHg)	193 ± 26 (144–253)	202 ± 25 (154–253)	181 ± 23 (144–225)	<0.01
Collected SBP/MET slope (mmHg/MET)	6.8 ± 2.9 (1.2–16.6)	6.7 ± 2.6 (2.5–15.0)	6.9 ± 3.3 (1.2–16.6)	0.72
ACSM SBP/MET slope (mmHg/MET)	5.5 ± 2.2 (1.1–4.5)	5.3 ± 1.9 (1.7–9.9)	5.7 ± 2.6 (1.2–14.5)	0.52
FRIEND SBP/MET slope (mmHg/MET)	6.0 ± 2.5 (1.0–14.8)	5.7 ± 2.1 (1.8–11.6)	6.4 ± 2.9 (1.0–14.8)	0.29

Data presented as mean ± SD (range). *P*-values represent comparisons between male and female participants using an unpaired *t*-test or Fisher’s exact test.

The clinical utility of workload-indexed peak SBP responses during exercise was established in a large cohort of 7542 male veterans^4^; however, subsequent studies using the SBP/MET slope derived from the ACSM equation have been much smaller (<75 participants).^9^ These smaller studies would be expected to have greater variability in the sampling distribution. Contributing to this variability is the fact that the ACSM equation does not account for factors aside from external workload, such as age and sex.^5^ The FRIEND equation accounts for these factors and has been shown to be a better predictor of VO_2_peak compared with the ACSM equation.^10^ As shown in the present data, and those derived from patients with coronary artery disease,^10^ the ACSM equation overestimates VO_2_peak. As a result, the ACSM equation tends to underestimate the SBP/MET slope and identified a smaller number of individuals with a slope >9 mmHg/MET. The FRIEND equation produced results that did not differ from collected measures, but both predictive equations resulted in low sensitivity and thus had greater numbers of false negative tests.

Overall, this study demonstrates the limitations of relying on the ACSM equation for estimating VO_2_peak during a maximal treadmill exercise test and calculating the SBP/MET slope. As the current high-risk SBP/MET slope of >9 mmHg/MET is based on the ACSM equation, future work is necessary to establish risk ranges using the FRIEND equation or, better yet, direct measures of VO_2_peak.

## Data Availability

The data that support this article are available from the corresponding author upon reasonable request.

## References

[1] Murray CJL , Aravkin AY, Zheng P, Abbafati C, Abbas KM, Abbasi-Kangevari M, et al Global burden of 87 risk factors in 204 countries and territories, 1990–2019: a systematic analysis for the Global Burden of Disease Study 2019. Lancet 2020;396:1223–1249. 33069327 10.1016/S0140-6736(20)30752-2 PMC7566194

[2] Schultz MG , Otahal P, Cleland VJ, Blizzard L, Marwick TH, Sharman JE. Exercise-induced hypertension, cardiovascular events, and mortality in patients undergoing exercise stress testing: a systematic review and meta-analysis. Am J Hypertens 2013;26:357–366. 23382486 10.1093/ajh/hps053

[3] Carlén A , Eklund G, Andersson A, Carlhäll CJ, Ekström M, Hedman K. Systolic blood pressure response to exercise in endurance athletes in relation to oxygen uptake, work rate and normative values. J Cardiovasc Dev Dis 2022;9:227. 35877589 10.3390/jcdd9070227 PMC9317915

[4] Hedman K , Cauwenberghs N, Christle JW, Kuznetsova T, Haddad F, Myers J. Workload-indexed blood pressure response is superior to peak systolic blood pressure in predicting all-cause mortality. Eur J Prev Cardiol 2020;27:978–987. 31564136 10.1177/2047487319877268

[5] Amercian College of Sports Medicine . ACSM’s Guidelines for Exercise Testing and Prescription. 10th ed. Philadelphia, PA: Wolters Kluwer Health; 2018.

[6] Bauer P , Kraushaar L, Dörr O, Nef H, Hamm CW, Most A. Sex differences in workload-indexed blood pressure response and vascular function among professional athletes and their utility for clinical exercise testing. Eur J Appl Physiol 2021;121:1859–1869. 33709207 10.1007/s00421-021-04656-x PMC8192366

[7] Bauer P , Kraushaar L, Dörr O, Nef H, Hamm CW, Most A. Workload-indexed blood pressure response to a maximum exercise test among professional indoor athletes. Eur J Prev Cardiol 2021;28:1487–1494. 33611510 10.1177/2047487320922043

[8] Myers J , Kaminsky LA, Lima R, Christle JW, Ashley E, Arena R. A reference equation for normal standards for VO2 max: analysis from the fitness registry and the importance of exercise national database (FRIEND Registry). Prog Cardiovasc Dis 2017;60:21–29. 28377168 10.1016/j.pcad.2017.03.002

[9] Currie KD , Floras JS, La Gerche A, Goodman JM. Exercise blood pressure guidelines: time to re-evaluate what is normal and exaggerated? Sports Med 2018;48:1763–1771. 29574665 10.1007/s40279-018-0900-x

[10] Jang WY , Kang DO, Park Y, Lee J, Kim W, Choi JY, et al Validation of FRIEND and ACSM equations for cardiorespiratory fitness: comparison to direct measurement in CAD patients. J Clin Med 2020;9:1889. 32560313 10.3390/jcm9061889 PMC7356312

